# The Impact of Seasonal and Meteorological Factors on Microorganisms Present in Knee Joint Effusions Among Patients with Rheumatoid Arthritis

**DOI:** 10.3390/ph19030347

**Published:** 2026-02-24

**Authors:** Hong Xiong, Shiyu Ji, Qian Ding, Yong Zhou, Xueming Yao, Yizhun Zhu

**Affiliations:** 1 Faculty of Chinese Medicine, State Key Laboratory of Quality Research in Chinese Medicine, Macau University of Science and Technology, Macau SAR 999078, China; xh13984160097@163.com; 2 Department of Rheumatology and Immunology, The Second Affiliated Hospital of Guizhou University of Traditional Chinese Medicine, Guiyang 550000, China; 3 School of Pharmacy, Laboratory of Drug Discovery from Natural Resources and Industrialization, State Key Laboratory of Quality Research in Chinese Medicine, Macau University of Science and Technology, Macau SAR 999078, China

**Keywords:** rheumatoid arthritis, knee joint effusions, climatic lag, 16S rRNA high-throughput sequencing, Distributed Lag Non-linear Model

## Abstract

**Background/Objectives:** Rheumatoid arthritis (RA) is a chronic autoimmune disorder characterized by persistent synovial inflammation and vascular abnormalities. Emerging evidence suggests that dysbiosis of the microbiome contributes to the pathogenesis of this disease, while seasonal and meteorological variations represent significant factors influencing microbial community dynamics. However, the specific pathological mechanisms mediated by microbial populations within knee joint effusions of RA patients remain poorly elucidated. The present study employs 16S rRNA high-throughput sequencing technology to characterize seasonal variation patterns affecting microbial communities in knee joint effusions of RA patients and to investigate the relationship between microbial community structures and climatic lag effects. **Methods:** Microbial communities in knee joint effusion samples obtained from RA patients were analyzed using 16S rRNA high-throughput sequencing methodologies. A Distributed Lag Non-linear Model (DLNM) was applied to quantify the delayed effects of climatic variables on microbial community composition. The correlation patterns between meteorological parameters and community structure were elucidated through the integration of ridge regression and redundancy analysis (RDA). Preliminary identification of potential biomarkers was conducted using random forest algorithms. **Results**: According to research findings, the microbial composition of knee joint effusions in RA patients shows seasonal fluctuation patterns that are compatible with those seen in RA patients, even though there is no discernible seasonal change in β-diversity. Compared with samples obtained during other seasons, spring specimens exhibited significantly elevated relative abundances of both beneficial microorganisms and opportunistic pathogenic taxa. Random forest modeling identified *Escherichia-Shigella* and *Curtobacterium* as preliminary candidate biomarkers; however, external validation is required to establish their specificity as disease indicators. Further analysis revealed that although short-term meteorological fluctuations exert minimal influence on overall microbial diversity, specific alterations in mean wind speed (MWS) and relative humidity (RH) drive compositional changes in the microbial community, manifested as rapid responses from dominant bacterial taxa and compensatory buffering effects from rare taxa. **Conclusions:** This study suggests that the synovial cavity microbiota in RA patients may exhibit seasonal variation patterns that are statistically associated with environmental parameters, particularly humidity and temperature. Due to the inherent limitations of the cross-sectional study design, the preliminary candidate biomarkers identified herein require validation through external cohorts. Additional investigations incorporating healthy controls and osteoarthritis (OA) cohorts are necessary to confirm specificity and to elucidate the therapeutic potential of these microbial targets for RA microbiome interventions. Currently, insufficient evidence exists to establish causal relationships among microbial populations, joint pathology, and climatic factors. Longitudinal cohort studies are imperative to validate the temporal dynamics and clinical significance of these associations.

## 1. Introduction

The human body harbors a greater number of bacterial, viral, fungal, and yeast cells than human somatic cells. These microorganisms colonize various internal organs and physiological systems, including the circulatory and nervous systems, as well as external body surfaces such as the skin, oral cavity, gastrointestinal tract, respiratory tract, and urogenital tract [1,2]. Human health depends upon the maintenance of a stable equilibrium between the host and its microbial inhabitants; perturbations in this homeostatic balance influence numerous physiological processes [3]. Accumulating evidence in recent years has demonstrated that, in addition to genetic determinants, the composition and functional capacity of the microbiota are modulated by seasonal variations, meteorological parameters, environmental factors, lifestyle choices, and dietary preferences [4,5]. Acute onset of rheumatoid arthritis (RA) exhibits increased incidence during spring, whereas insidious onset is more prevalent during autumn. The rates of acute and insidious onset are generally comparable during summer and winter [6,7]. Furthermore, advancing age and alterations in environmental exposure substantially influence microbial diversity, community composition, and metabolic activity [8,9].

According to conventional understanding, the joint cavity was considered a sterile environment. However, recent investigations employing 16S rRNA high-throughput sequencing methodologies have enabled the detection of microbial DNA in synovial fluid and synovial tissue specimens obtained from RA patients. These microbial communities are predominantly composed of the phyla *Firmicutes* and *Proteobacteria* [10]. Of greater significance, these microbial constituents may actively participate in the pathological processes culminating in joint structural damage, rather than merely representing secondary consequences of inflammatory processes. Previous investigations have demonstrated that intra-articular microbial components may induce the acquisition of an invasive phenotype in synovial fibroblasts through activation of Toll-like receptors. This contributes to the degenerative process of cartilage degradation by promoting the release of matrix metalloproteinases and stimulating synovial proliferation [11,12]. Furthermore, metabolites derived from gut microbiota, such as short-chain fatty acids, may influence osteoclast differentiation and bone erosion through modulation of the RANKL/OPG axis [13,14]. To advance our understanding of RA pathophysiology, it is imperative to investigate the relationships among joint microbiota, autoantigen production, synovial fibroblast activation, and bone remodeling. The host environment may exert substantial influence in regulating microbial community composition. Epidemiological data indicate that RA disease activity and joint pain intensity exhibit pronounced seasonal variations, particularly during periods of reduced atmospheric pressure, elevated humidity and temperature fluctuations, when patient symptoms frequently deteriorate substantially [15,16]. From the perspective of microbial ecology, the host-associated microbiome exhibits considerable sensitivity to meteorological variables, including temperature, humidity, and light exposure [17]. Environmental alterations may indirectly influence the local microenvironment within joints in addition to modifying the host immunological state. However, direct experimental evidence is currently insufficient to determine whether seasonal variations select specific microbial communities through modification of synovial fluid physicochemical properties or whether they directly alter microbial composition within joint cavities. Further investigation is required to validate these hypotheses.

The present study employed 16S rRNA high-throughput sequencing methodologies to characterize the microbial community structure in knee joint effusions obtained from 72 RA patients. The objective was to investigate potential correlations between seasonal variations in microbial communities and environmental parameters. This investigation provides novel insights for optimizing RA therapeutic strategies and establishing a weather-sensitive microbiological biomarker system, with significant implications for improving clinical outcomes in RA patients and developing climate-sensitive healthcare protocols.

## 2. Results

### 2.1. Analysis of Sequence Depth and Rank Abundance

The raw sequence reads from the 72 sequenced samples ranged from 78,028 to 81,968, whereas effective sequences ranged from 48,359 to 72,816. A total of 176 amplicon sequence variants (ASVs) were identified across all four seasons; ASV classification analysis revealed 2394, 2230, 1893, and 1974 ASVs in spring, summer, autumn, and winter, respectively (see Supplementary Materials). The rank–abundance curves exhibited a characteristic long-tail distribution pattern, with relative abundance declining rapidly as ASV rank increased within each group. The summer season demonstrated the highest mean relative abundance across all ASV ranks, whereas spring exhibited the lowest mean value, indicating comparatively reduced relative abundance. These patterns reflect differential species richness and distribution of dominant taxa among groups, illustrating compositional differences in microbial communities across seasonal categories. The curves demonstrate that the quantity and relative abundance of joint effusion microbial communities approach asymptotic values as sequencing depth and corresponding rank values increase, indicating that adequate sequencing depth has captured a representative level of community richness (see Supplementary Materials).

### 2.2. Microbial Community Structure

Based on species annotation results, cumulative bar charts illustrating relative taxonomic abundance were generated by selecting the 15 most abundant phyla from each sample. Across all four seasons, more than 99% of total bacterial abundance was attributable to the phyla *Proteobacteria*, *Bacteroidetes*, *Actinobacteria*, and *Firmicutes* (see Supplementary Materials). Specifically, *Bacteroidetes* demonstrated reduced relative abundance during summer, whereas *Actinobacteria* and *Proteobacteria* exhibited lower relative abundance during spring compared with other seasons. *Firmicutes* demonstrated elevated relative abundance during summer. Kruskal–Wallis testing revealed that *Verrucomicrobiota* was the sole phylum exhibiting significant seasonal variation (*p* < 0.05).

### 2.3. Analysis of α and β-Diversity

**Figure 1:**
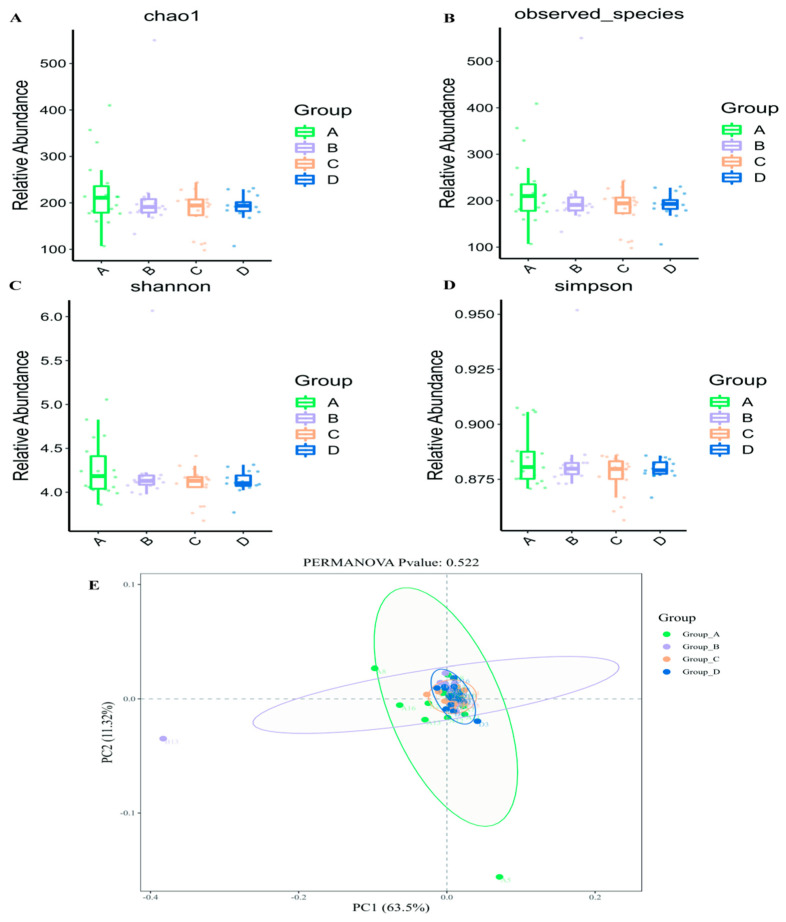
Analysis of α and β-diversity ((**A**) Chao 1 index; (**B**) Observed Categories index; (**C**) Shannon index; (**D**) Simpson index; (**E**) PCA with β-diversity of microorganisms).

Temporal comparison of α-diversity indices revealed seasonal patterns in microbial diversity and abundance in knee joint effusions from RA patients (Figure 1A–D). Microbial species richness and abundance demonstrated relative increases during spring, whereas other seasons exhibited varying degrees of reduction. Summer and winter displayed comparable patterns of microbial species richness, whereas autumn demonstrated comparatively diminished species richness.

Principal component analysis (PCA) based on Binary Jaccard distances was performed on seasonal samples (Figure 1E). Principal component 1 (PC1) accounted for 63.5% of variance, whereas principal component 2 (PC2) contributed 11.32%. The four sample groups exhibited no discernible regional overlap or clustering pattern. The absence of distinct clustering zones within sample groups indicates substantial similarity in microbial community composition among different groups. Additional permutation multivariate analysis of variance (PERMANOVA) testing for intergroup differences revealed no statistically significant differences (*p* = 0.522; *p* > 0.05). These findings indicate that, despite variations in microbial abundance (α-diversity), the overall species composition structure (β-diversity) of the joint effusion microbiota remains largely consistent across seasons. This pattern of “consistent overall framework with localized abundance alterations” suggests that seasonal influences primarily affect the relative abundance of specific taxa rather than inducing fundamental alterations in community membership. However, longitudinal investigations with expanded sample sizes are required to validate whether this consistency represents genuine ecological stability.

### 2.4. Seasonal Variations in Microbial Communities: Analysis of Opportunistic Pathogens and Beneficial Bacteria

**Figure 2:**
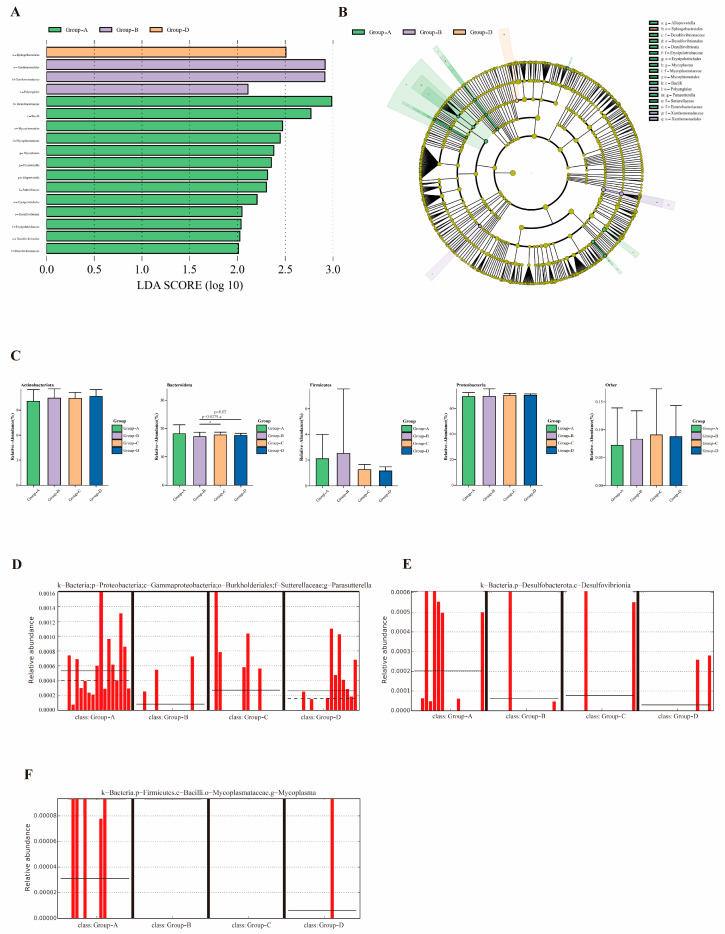
Indicative seasonal fluctuations in the microbiome composition of knee joint effusions. ((**A**) Differential species score plot (LDA SCORE indicates the magnitude of the difference contribution; the larger the absolute value, the greater the difference contribution); (**B**) Cladogram annotation of the differential species (node diameter is proportional to relative abundance); (**C**) Four microbiome groups with notable differences (the vertical axis shows the relative abundance values of the species, while different colors correspond to distinct sample groups; The relative abundance of *Bacteroidota* in different groups was significantly different from that in Group-B (* *p* < 0.05); (**D**–**F**) Relative abundance histogram (each bar shows the relative abundance of each samples within each group; the solid line is the mean relative abundance and the dotted line shows the median relative abundance)).

At the genus level, three distinct bacterial groups were identified: *Mycoplasma*, *Parasutterella*, and *Alloprevotella* demonstrated enrichment during spring. At the class level, two differentially abundant taxa were identified: *Bacilli* and *Desulfovibrionia* exhibited increased abundance during spring. At the order level, six differentially abundant taxa were identified: *Mycoplasmatales*, *Erysipelotrichales* and *Desulfovibrionales* demonstrated enrichment during spring; *Xanthomonadales* and *Polyangiales* exhibited enrichment during summer; and *Sphingobacteriales* demonstrated enrichment during winter. At the family level, six differentially abundant taxa were identified: *Enterobacteriaceae*, *Mycoplasmataceae*, *Sutterellaceae*, and *Erysipelotrichaceae* demonstrated enrichment during spring, whereas *Xanthomonadaceae* exhibited enrichment during summer (Figure 2A,B). The Wilcoxon rank-sum test was employed to compare relative abundances among the four groups at the phylum level. *Proteobacteria* represented the predominant bacterial group across all sample groups. *Actinobacteria* and *Firmicutes* were consistently present but at relatively low abundances. *Bacteroidetes* exhibited the most pronounced intergroup differences, suggesting a potential role in disease activity or group classification. The low-abundance “Other” category contributed minimally to overall community composition (Figure 2C).

Two beneficial bacterial genera in knee joint effusions, *Parasutterella* and *Alloprevotella*, exhibited consistent seasonal variation patterns (Figure 2D,E), with higher mean relative abundances during spring compared with other seasons. The genus *Mycoplasma*, which encompasses opportunistic pathogens in knee joint effusions, demonstrated elevated prevalence during spring compared with other seasons (Figure 2F).

### 2.5. Random Forest Intergroup Prediction

**Figure 3:**
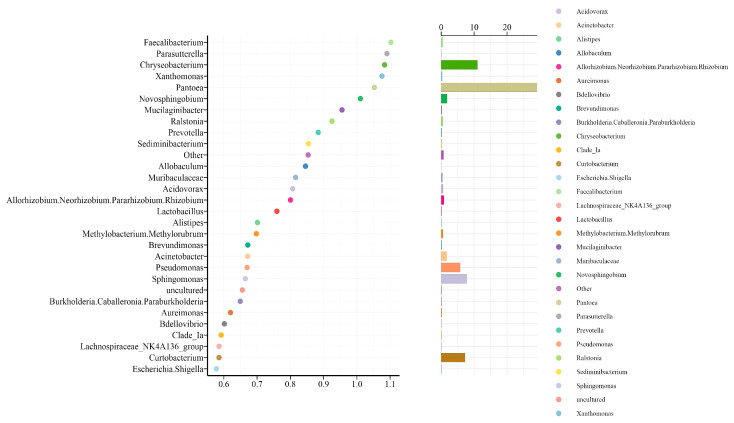
Plot of random forest intergroup prediction. (The vertical axis shows species names arranged by importance, and the horizontal axis shows the important metric).

A random forest model constructed at the genus level identified thirty microbial genera as significant contributors to the classification model (Figure 3). Among these, *Escherichia-Shigella* (importance score: 1.1) and *Curtobacterium* (importance score: 1.0) emerged as the primary markers dominating sample classification. Bubble size analysis identified *Escherichia-Shigella* as a fundamental taxonomic marker, whereas *Curtobacterium* represents a likely low-abundance but highly specialized marker. The composite designation *Burkholderia-Caballeronia-Paraburkholderia* refers to closely related, taxonomically similar genera that should be considered as a functional unit in research contexts. The differential importance between *Faecalibacterium* (low importance) and *Lachnospiraceae* (medium-high importance) reflects the study’s focus on pathological states rather than healthy conditions. The substantial relevance of environmental bacteria such as *Curtobacterium* and *Sphingomonas* (sphingomyelin-degrading bacteria) suggests potential environmental influences on the samples.

### 2.6. Effects of Meteorological Lag

In environmental epidemiology, the biological latency period of the exposure factor is typically employed to determine the lag window for the Distributed Lag Non-linear Model (DLNM). To establish distinct lag intervals, the present study integrated commonly employed time intervals with the physical characteristics of individual meteorological parameters and the anticipated response time of joint cavity microbiota (Table 1). Lag times for each meteorological variable were determined using the DLNM: apparent temperature (AT), 0–2 days; daily temperature range (DTR), 5 days; relative humidity (RH), 3–5 days; mean wind speed (MWS), 2–13 days; and cloud cover (CC), 13–14 days. Additionally, mean meteorological data were calculated for the lag days corresponding to the sampling dates of the 72 samples.

### 2.7. α-Diversity and Meteorological Factors

**Figure 4:**
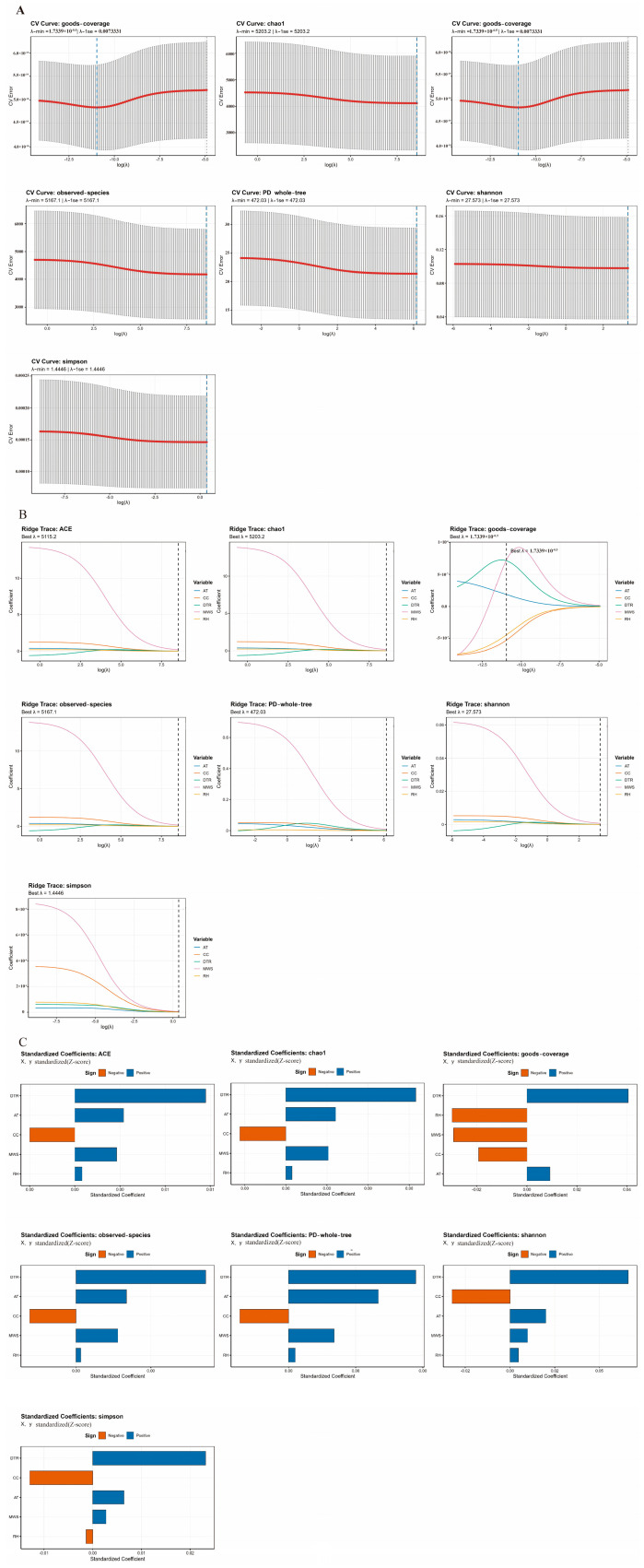
(**A**) CV curves for different α-diversity indices in LASSO models (the grey bars show ±1 standard error, and the red curve shows the mean CV error over folds. The chosen tuning parameter (λ_min and/or λ_1se, as stated above each panel) is indicated by the vertical dashed line. This parameter is utilized to specify the ideal penalization level for interpretation downstream). (**B**) Trajectories of ridge regression coefficients for different α-diversity indices (The estimated coefficient of one predictor (AT, CC, DTR, MWS, and RH) as a function of log(λ) is represented by each colored line. Coefficients gradually shrink toward zero as λ rises, demonstrating the predictors’ stability and relative contribution under more severe penalization. The ideal λ for obtaining final ridge estimates is shown by the vertical dashed line). (**C**) Bar charts of standardized regression coefficients for different α-diversity indices (The z-score standardization of the predictors (X) and outcomes (Y) allowed for a direct comparison of effect sizes between variables. Larger absolute coefficients indicate bigger contributions to the relevant α-diversity metric. Bars show the magnitude and direction of relationship, with orange denoting negative impacts and blue denoting positive effects).

K-fold cross-validation (CV) was employed to identify the penalty parameter λ during construction of LASSO regression models using lagged meteorological data (AT, DTR, RH, CC, and MWS) as independent variables and various α-diversity indices as dependent variables (Figure 4A). The CV error curves for the ACE, Chao1, observed_species, PD_whole_tree, Shannon, and Simpson indices demonstrated only marginal decline with increasing log(λ). Both λ_min and λ_1se were located at the right extremity of the search range and exhibited substantial overlap. This indicates that minimal models with coefficients approaching zero demonstrate the lowest prediction error under strong regularization. The inclusion of lagged climatic data did not substantially improve prediction of these diversity indices, suggesting that their linear influence on overall microbial richness and evenness is modest. In contrast, the CV error curve for the goods_coverage index exhibited a modest U-shaped pattern, with minimum error occurring at smaller λ values and gradually increasing as regularization intensified. Although the error associated with λ_min was lower than that for the majority of λ values, it differed only marginally from λ_1se, which falls within one standard error. Based on the one-standard-error criterion, λ_1se was selected for construction of the final model. This suggests that lagged meteorological factors provide only limited additional explanatory power overall and demonstrate a discernible but weak correlation signal with microbial community coverage. The trace plots demonstrate consistent trends in regression coefficients across various meteorological variables with respect to the regularization parameter λ for the combined effect of meteorological factors on knee joint effusion microbiological diversity and structure. However, the stability and absolute magnitudes of these coefficients varied depending on the diversity metric.

Ridge regression coefficient trajectories were also presented to evaluate the robust contributions of various lagged meteorological variables to each α-diversity index, with the optimal penalty parameter determined through CV (Figure 4B). Generally, MWS demonstrated comparatively substantial positive regression coefficients for the ACE, Chao1, observed_species, PD_whole_tree, Shannon, and Simpson indices when log(λ) was small (mild regularization). RH coefficients approximated zero, DTR coefficients were marginally above zero, and CC and AT coefficients were modest and positive. As λ increased, the coefficients for all meteorological variables declined monotonically and rapidly approached zero. At the optimal λ selected through CV, the absolute values of coefficients for each of the five lagged meteorological variables approximated zero. This finding indicates that, after substantially constraining model complexity to prevent overfitting, the linear contribution of any single lagged meteorological variable to the aforementioned diversity indices is minimal, and its effect is rapidly attenuated under regularization constraints.

Standardized regression analyses examining the association between microbial community diversity and meteorological lag variables were also performed (both X and Y variables were processed using Z-scores; Figure 4C). Overall, DTR demonstrated the highest absolute standardized coefficient across species richness and evenness indices, including ACE, Chao1, observed_species, Shannon, and Simpson, followed by AT. MWS and RH coefficients approximated zero, whereas CC coefficients were predominantly moderately negative. This implies that, although the directionality varies among different meteorological variables, the linear effects of lagged meteorological conditions on α-diversity are generally weak: elevated CC was associated with marginally reduced diversity, whereas elevated DTR and AT were associated with slightly increased species richness and evenness.

For the phylogenetic diversity index PD_whole_tree, DTR demonstrated the highest standardized coefficient, followed by AT. MWS also demonstrated a positive influence. Conversely, RH approximated zero and CC exhibited a negative coefficient, indicating that lagged temperature levels and DTR demonstrate comparatively greater sensitivity in their correlation with the phylogenetic breadth of microbial communities.

In contrast to the aforementioned indices, the standardized coefficients for goods_coverage demonstrated distinct positive and negative correlations: DTR and AT exhibited positive values, whereas MWS, RH, and CC exhibited negative coefficients. Among these, the negative effect of RH and the positive effect of DTR demonstrated comparatively high absolute values. This finding suggests that, after adjusting for other climatic parameters, elevated AT and DTR are associated with modest increases in microbial community coverage, whereas elevated MWS, RH, and CC are associated with reduced coverage. Goods_coverage demonstrates comparatively elevated sensitivity to variations in humidity and temperature.

Collectively, the results from CV, ridge plots, and standardized coefficients indicate that lagged meteorological variables exert relatively modest overall influence on the α-diversity of knee joint effusion microbiota at the temporal scale and exposure levels investigated in this study. Conversely, RH and CC demonstrate distinctly negative associations with microbial coverage, whereas CC and AT exhibit comparatively consistent positive trends across multiple diversity indices. These findings suggest that the microbial diversity of knee joint effusions is resilient to transient meteorological fluctuations. More refined indicators, such as coverage, may exhibit enhanced sensitivity to specific climatic factors, particularly RH and CC.

### 2.8. β-Diversity and Meteorological Factors

**Figure 5:**
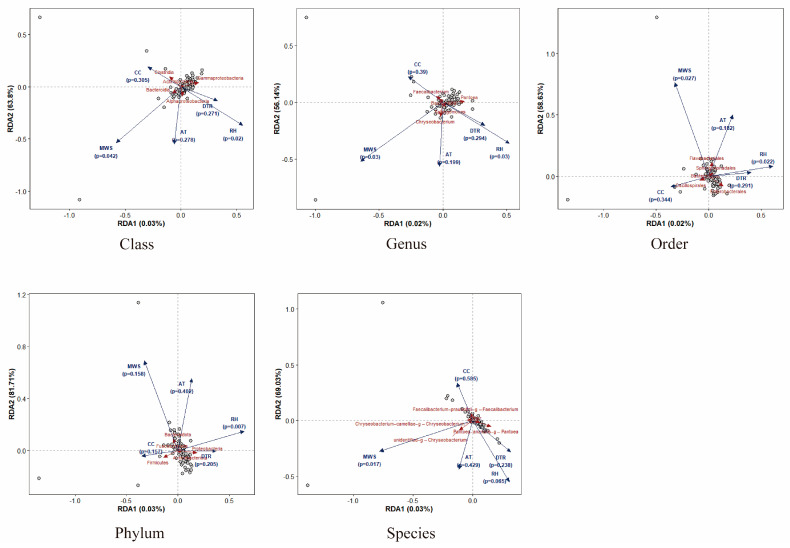
Diagrams of RDA at different levels. (RDA biplots that illustrate the connections between environmental factors and the makeup of microbial communities at five different taxonomic levels. Individual samples are represented by black open circles, and the taxa (or taxonomic groups) that are most strongly contributing to the ordination are shown by red labels. Explanatory variables (AT, CC, DTR, MWS, and RH) are indicated by blue arrows; the direction of the arrows shows which way the values are growing, and the length of the arrows shows how strongly the variables are correlated with the ordination structure. The estimated location of taxa or samples along an arrow represents how closely they are related to that variable. The proportion of constrained variance that RDA1 and RDA2 account for is indicated by the percentages on the RDA axes. The statistical relevance of each environmental element in explaining community variance is indicated by the *p* values (located next to each arrow) that were derived from permutation testing).

Redundancy analysis (RDA) was performed at the class, genus, order, phylum, and species levels to examine the effects of lagged meteorological variables (AT, DTR, RH, CC, MWS) on β-diversity of knee joint effusion microbiomes (Figure 5). The two constrained axes, RDA1 and RDA2, collectively explained the majority of variance (56.1–81.7%) associated with climatic variables. RH and MWS emerged as the most consistent drivers across all taxonomic levels. The ordination vectors for RH and MWS achieved statistical significance at the class, genus, and order levels (class: RH *p* = 0.02, MWS *p* = 0.042; genus: RH and MWS both *p* = 0.03; order: RH *p* = 0.022, MWS *p* = 0.027), whereas *p*-values for AT, DTR, and CC all exceeded 0.05. The vector directions were essentially opposite, indicating that elevated humidity and reduced wind velocity simultaneously drive microbial community composition along the same environmental gradient: RH oriented toward the positive quadrant of RDA1, whereas MWS oriented toward the negative quadrant. Whereas certain prominent groups (such as *Gammaproteobacteria* and *Faecalibacterium*) aligned with the RH vector direction, indicating relative sensitivity to humidity and wind velocity changes, some wind-speed-related species clustered near the MWS vector extension. At the phylum level, only RH demonstrated a significant association (*p* = 0.007) with community structure, and its vector direction largely corresponded with the distribution patterns of phyla such as *Proteobacteria*. Conversely, MWS, AT, DTR, and CC did not achieve statistical significance. Species-level results were highly consistent with genus-level results: AT, DTR, and CC demonstrated no significant influence, RH exhibited a marginally significant trend (*p* = 0.065), and MWS remained significantly correlated (*p* = 0.017).

From an ecological perspective, these findings suggest that the microbial community of knee joint effusions may be influenced by meteorological conditions through multiple mechanisms. Enhanced temperature fluctuations and moderate wind velocities contribute to the maintenance and promotion of microbial diversity by improving microhabitat conditions and facilitating exchange and dissemination of microbial communities from diverse sources. Conversely, elevated humidity and cloud cover may reduce transpiration and light exposure, thereby limiting the competitive advantage of certain microbial communities and shifting community structure toward reduced evenness. Generally, RH and MWS emerge as important meteorological factors promoting diversity and richness in knee joint effusion microbiomes, whereas CC demonstrates inhibitory effects in most contexts. Although sensitivity varies, this pattern is evident in both richness-based and evenness-based metrics. This discovery provides important insights for predicting and managing microbial diversity under climate change scenarios, in addition to elucidating potential regulatory mechanisms of climatic variables on microbiome ecological processes in knee joint effusions.

In conclusion, RDA results demonstrate that lagged meteorological factors explain only partial variation in the β-diversity of knee joint effusion microbiota under the exposure range and temporal scale of this study; however, they still exert some influence on the direction of community structure changes. Among these, RH and MWS were frequently identified across various taxonomic levels, demonstrating an inverse gradient with shifts in community composition and emerging as the primary environmental factors influencing structural variations in microbial communities. Conversely, the contributions of AT, DTR, and CC to community structure were minimal. This is consistent with the results of the preceding α-diversity analysis, indicating that although variations in RH and MWS can still induce minor rearrangements in microbial composition, short-term climatic fluctuations exert limited influence on overall microbial diversity.

## 3. Discussion

Recent investigations into microbial ecology have established direct relationships between microbial homeostasis and host processes including pathogen defense, development, immunological regulation, and metabolic function. Endogenous and exogenous hormonal interactions can modulate microbial communities, thereby influencing the ecological equilibrium of the host organism. Alterations in the composition of the human microbiome may precipitate a loss of immunological tolerance, suggesting that microbial dysbiosis may play a substantial role in the pathogenesis of autoimmune disorders [21,22]. RA is a systemic inflammatory disorder characterized primarily by persistent, progressive joint destruction. Research indicates that alterations in gut microbiota composition during the immunological initiation phase may trigger joint inflammation [23]. Controlled studies have demonstrated that management of periodontal pathogen infections in RA patients substantially ameliorates arthritic symptoms [24], suggesting that oral bacteria may contribute to RA pathogenesis through immune system activation. Given that the gut microbiota exhibits immunoregulatory functions and influences RA progression, microbe-host immunological interactions have emerged as a critical perspective for understanding RA pathogenesis. However, the characteristics of the local microbial community within the joint cavity—the immediate site of RA pathology—and their relationship with disease activity remain poorly characterized. The present study employs 16S rRNA high-throughput sequencing technology to characterize the microbial composition of knee joint effusions in RA patients, thereby offering novel insights into RA research from a local microbiome perspective.

The findings of this study indicate that the microbial diversity and community structure of knee joint effusions in RA patients appear to vary seasonally, though further validation is warranted. Preliminary analysis indicates that compared to other seasons, spring exhibits relatively greater fluctuations in species richness and diversity. The diversity of the human gut microbiota is commonly influenced by multiple endogenous and exogenous factors, including season, diet, and medication [25]. The present study demonstrates that seasonal conditions similarly influence microbial diversity in knee joint effusions of RA patients. *Firmicutes* and *Bacteroidetes* constitute approximately 90% of the relative abundance of the human gut microbiome, whereas *Actinobacteria* and *Proteobacteria* account for less than 10%. This gut microbial structure is essential for maintaining intestinal homeostasis [26]. When gut microbiota dysbiosis occurs, intestinal bacteria may function as inflammatory mediators, affecting mucosal and systemic immunological activities and thereby precipitating immune-mediated disorders [27]. With regard to microbial community composition in knee joint effusions of RA patients, this study identified four dominant phyla: *Proteobacteria* (70%), *Bacteroidetes* (17.3%), *Actinobacteria* (10.6%), and *Firmicutes* (1.5%). Alterations in the dominant phyla in RA knee effusions were attributable primarily to changes in abundance rather than composition. Die Yu et al. [28] reported that Ascomycota and *Actinobacteria* were more prevalent in healthy individuals, whereas *Bacteroidetes* and *Proteobacteria* were more abundant in RA patients. Investigations elucidating functional variations in the human gut microbiota have demonstrated that *Proteobacteria* constitute a major reservoir of variable genes that may serve as biomarkers for dysregulation of host homeostasis and inflammation in healthy hosts [29]. The predominance of *Proteobacteria* in knee joint effusions of RA patients suggests a potential association between this phylum and RA disease activity. At the phylum level, *Verrucomicrobiota* exhibited relatively pronounced seasonal variation in abundance compared to other taxa examined. This phylum was detected in knee joint effusions, consistent with its emerging characterization in gastrointestinal contexts following the discovery of *Akkermansia muciniphila* in 2004 [30]. Additionally, both p_*Verrucomicrobiota* and *g_Akkermansia* showed positive correlations with elevated levels of long-chain fatty acids (9,12-octadecadienoic acid and 10Z-nonadecenoic acid) in RA patients [31,32,33], raising the possibility of functional links between *Verrucomicrobiota* and host lipid metabolism in joint microenvironments. By reducing the amount of *Verrucomicrobiota* in the gut, which in turn inhibits the activity of its encoded dihydrofolate reductase (DHFR), methotrexate (MTX) raises the concentration of methotrexate polyglutamate (MTX-PG) in host red blood cells and increases purine synthesis inhibition and immunosuppression, according to some studies [34]. At the community level, PERMANOVA revealed no significant seasonal differences, despite indicator species analysis suggesting seasonal preferences for individual taxa. This finding implies that the overall community structure remains largely stable throughout the year, despite seasonal variations in the abundance of specific taxa. Consequently, the available data are insufficient to establish seasonal variations in the general community structure. This observation provides a critical perspective for understanding the relationship between seasonal fluctuations and the microbiota of RA patients and may guide future investigations. Analysis of samples from multiple seasons revealed seasonal fluctuations in the microbiota at several taxonomic levels. Seasonal rhythms of microbial communities have been extensively documented across diverse habitats. For example, longitudinal research in East Lake, Wuhan, demonstrated notable seasonal differences in planktonic bacterial community structure [35]. Similarly, investigations of phyllosphere microbiota revealed distinct variations in microbial richness and functional capacity (including sulfur and glucose metabolism) between dry and wet seasons [36]. These findings corroborate our observation that season-specific microbial enrichment represents an adaptive response to cyclical environmental parameters such as humidity and temperature, rather than a stochastic phenomenon. Notably, the springtime enrichment of *Mycoplasma* observed in this study is consistent with findings in animal hosts. For instance, research has demonstrated that *Mycoplasma* abundance in the respiratory tracts of healthy desert tortoises similarly peaks during spring [37]. This study suggests that, depending on the host species, geographical location, and specific ecological niche, seasonal dynamics of microorganisms within the same taxon may exhibit considerable specificity. Furthermore, *Mycoplasma* infection markedly increases the production of synovial interleukin-1β (IL-1β), interleukin-6 (IL-6), interleukin-17 (IL-17), and prostaglandin E2 (PGE2), while downregulating interleukin-10 (IL-10), thereby contributing to the establishment of an inflammatory joint environment [38]. As a prominent phylum within host-associated microbiomes, variations in the abundance and composition of *Bacteroidetes* are frequently directly associated with the host’s health status. Additionally, microbial communities enriched during spring, such as *Bacillales* and *Paracellulomonas*, may harbor functional genes associated with specific seasonal metabolic requirements. This observation is consistent with research on phyllosphere microbiomes. *Proteobacteria* play a crucial role in this system and demonstrate substantial environmental flexibility as a dominant phylum with cross-seasonal stability. The overall structural and functional resilience of the community is constituted by the stability of this “core microbiome” and the seasonally variable “environmentally responsive microbiome,” represented by *Bacteroidetes* [39]. This phenomenon is consistent with contemporary hypotheses regarding microbial community assembly [40]. This study identified the simultaneous enrichment of opportunistic pathogenic genera and potentially beneficial bacterial genera in knee joint effusions during spring. This finding suggests that spring may represent a critical period for local microbial ecological transitions within joints. Among these, the genera *Parasutterella* and *Alloprevotella*, which function as beneficial bacteria in the gastrointestinal tract, appear to confer benefits in knee joint effusions. According to published findings, *Alloprevotella* rava, a member of the *Alloprevotella* genus, possesses the capacity to produce short-chain fatty acids (SCFAs) through glycolysis, generating acetate and succinate [41]. The latter can function as a substrate for butyrate-producing bacteria through cross-feeding mechanisms, thereby indirectly promoting butyrate synthesis [42]. Although *Parasutterella* does not directly produce substantial quantities of SCFAs, it is tightly associated with host metabolic health [43,44]. Through analogous metabolites, their concentration in synovial fluid may exert regulatory effects on the intra-articular immune milieu, such as attenuating excessive inflammation. However, this study can only demonstrate alterations in relative abundance and cannot verify the actual metabolic activity of these taxonomic groups. In clinical practice, patients presenting with joint effusions may benefit from microbiological monitoring and intervention during spring.

Random forest classification modeling at the genus level identified *Escherichia-Shigella* and *Curtobacterium* as the two most predictive core biomarkers. *Escherichia-Shigella* may represent a novel diagnostic marker given its elevated abundance in osteoarthritis (OA), Sjögren’s syndrome (SS) and RA [45,46]. Research has demonstrated that whereas *Escherichia-Shigella* exhibits markedly elevated abundance in the feces of RA patients, butyrate-producing bacteria are substantially reduced. Fecal microbiota transplantation (FMT) or high-fiber dietary interventions can rectify this imbalance by decreasing the relative abundance of *Escherichia-Shigella* [47]. Notably, *Escherichia-Shigella* and *Pseudomonas* are significantly associated with urinary tract infections, diarrhea, and inflammatory bowel disease, suggesting potential involvement in host immunological modulation [48,49]. Conversely, decreased levels of *Faecalibacterium*, a crucial butyrate-producing bacterium in the healthy gut, have been extensively documented in numerous disease states, including Crohn’s disease, colorectal cancer, and metabolic syndrome [50,51]. In addition to its correlation with systemic inflammatory markers, *Escherichia-Shigella* can accelerate microstructural damage and bone erosion in subchondral bone by directly inducing local osteoclast production and activation through its metabolites, including lipopolysaccharide (LPS) [52,53]. Based on the springtime pathogen enrichment observed in this study, supplementation with specific prebiotics or anti-inflammatory nutrients (such as omega-3 polyunsaturated fatty acids and polyphenolic compounds) during spring or high-humidity periods may help suppress excessive proliferation of *Escherichia-Shigella* species while promoting balanced endogenous probiotic populations [54,55,56]. These intervention strategies, which focus on modulating the microbial community, may provide potential adjunctive support for preserving joint microstructural integrity by attenuating inflammatory cascades triggered by seasonal dysbiosis. As producers of SCFAs, members of the *Lachnospiraceae* family exhibit varying disease-associated patterns, indicating functional heterogeneity across different clinical contexts [51]. Other research has characterized *Curtobacterium* exclusively as an “environmentally associated low-abundance bacterium,” with no documentation of its strains, immunological characteristics, or functional role [57]. Given its low abundance, susceptibility to environmental contamination, and limitations in experimental control, *Curtobacterium* should not be considered a reliable biomarker.

To evaluate the lagged effects of climatic conditions on the α- and β-diversity of knee joint effusion microbiota and their ecological driving mechanisms, this study integrated DLNM with ridge regression and RDA. We found that whereas CC consistently exerted a negative influence on α-diversity, DTR and MWS represented the most favorable indicators. RH and MWS imposed strong directional selection pressures on community composition across five taxonomic levels: phylum, class, order, genus, and species within the *Proteobacteria* domain. DLNM results demonstrated that temperature signals exerted immediate to short-term effects on arthrobiont communities, with lag windows of 0–2 days for AT and 5 days for DTR. In contrast, MWS and CC exhibited lag times of 13–14 days, which closely corresponded to the long-range transport intervals (7–21 days) for airborne microorganisms reported in previous investigations [58]. Ridge regression CV curves demonstrated that even at elevated λ intervals, richness metrics (ACE, Chao1, and observed species) retained prediction accuracy, indicating statistical relationships with multiple climatic variables. Conversely, evenness metrics (Shannon, Simpson) converged at very low λ values, suggesting that a limited number of dominant factors, including DTR and MWS, account for the majority of their variability. This divergence most likely reflects differential responses of dominant species and rare taxa to environmental gradients: evenness metrics, which are dominated by the abundance of dominant species, exhibit more direct responses to important environmental factors (e.g., DTR, MWS), whereas richness metrics, which are driven by rare taxa, require the integration of multiple environmental factors for prediction. This “richness-stable, evenness-sensitive” pattern is consistent with general principles governing microbial community assembly and does not represent an anomalous observation [59]. The richness-evenness trade-off is a cross-ecosystem characteristic that enhances the generalizability of research findings, as evidenced by its documentation across marine, soil, and host microbiomes [60,61]. Interestingly, the optimal λ value for the PD_whole_tree index falls between these two extremes, suggesting that phylogenetic diversity may incorporate signals from both long-term dispersal (rare species) and short-term selection (dominant species). According to RDA analysis, the explanatory power of meteorological parameters was distributed primarily along the second ordination axis (RDA2) and increased with increasing taxonomic resolution. This is consistent with RDA1 residual variance representing dispersal limitations and RDA2 indicating environmental filtering [62,63]. The beneficial effects of RH for *Proteobacteria* at the phylum level are consistent with previous findings that “high humidity promotes Gram-negative bacterial bloodstream dissemination” [64]. At the genus level, DTR and RH promote *Pantoea* while inhibiting *Faecalibacterium*, indicating that temperature-humidity variations may reduce the colonization advantage of butyrate-producing bacteria through oxidative stress or short-chain fatty acid depletion [50]. The species-level strong correlation between *Pantoea ananatis* and RH demonstrates phylogenetically specialized niche differentiation of microbes to microclimate conditions, suggesting its fine-tuned regulation by environmental filtering [65,66]. The DLNM-ridge regression-RDA combined analysis revealed lagged relationships between climatic parameters (DTR, RH) and joint cavity microbial richness and *Proteobacteria* abundance, suggesting that environmental filtering may exert cumulative temporal effects [19]. This finding is consistent with previous research: low temperatures (≤5 °C) have been demonstrated to increase the incidence of prosthetic joint infection (PJI) following surgery; joint cavities are not sterile environments; and *Proteobacteria* are significantly enriched in OA patients [67,68]. These findings support the hypothesis that weather conditions may influence opportunistic pathogen abundance, which in turn may modulate joint microbiomes. In the context of global climate change, an increase in the frequency of extreme weather events may exacerbate the burden of infectious diseases and further disturb microbiome homeostasis by increasing DTR and RH fluctuations. Due to the cross-sectional design employed in this investigation, these relationships represent merely statistical associations of time–exposure–response relationships and cannot be used to establish causality or support clinical “early warning windows.” Future longitudinal cohort studies integrating metagenomic functional analysis and host immune biomarkers (e.g., IL-6, CRP) are required to validate causal pathways in weather–microbiome–host interactions.

Overall, the knee joint effusion microbiome of RA patients exhibits a noticeable decrease in beneficial bacteria and a marked increase in opportunistic pathogens. Significant seasonal preferences influenced by the four seasons were identified in the knee joint effusion microbiota of RA patients. This study identified distinct genetic features in the joint cavity microbiota of RA patients that may represent targets for microbe-directed therapeutic interventions. Nevertheless, these findings currently represent merely associations, and additional functional validation and clinical trials are required to establish their validity. Drawing upon the “lag-diversity-hierarchical response” analytical framework, this study offers a preliminary understanding of the relationship between joint cavity microbiota and climatic parameters.

This study is subject to the following limitations:

(1) Limitations of Representativeness and Study Design: This investigation constitutes a cross-sectional exploratory cohort with a single center and limited sample size (*n* = 72). The wide age range, unbalanced gender ratio, and absence of controls with non-inflammatory arthritis constrain the generalizability of conclusions and disease-specific validation. Although our findings suggest indicative seasonal fluctuations, validation in larger cohorts using quantitative real-time PCR (qRT-PCR) is warranted in future research. The regional lifestyle and humid plateau environment of a single city may introduce spatially specific confounding factors; therefore, multicenter studies encompassing diverse climatic conditions are required for validation.

(2) Technical Approaches and Data Constraints: This investigation collected only relative abundance data for taxonomic groups; metabolites including short-chain fatty acids and cytokine levels were not measured. Important environmental factors, including ultraviolet radiation, PM2.5/PM10, and daily real-time meteorological data, were also not incorporated. Although low-biomass samples were decontaminated using bioinformatics approaches, findings pertain primarily to dominant taxa. Individual variations in specific medication regimens could exert confounding effects on microbiome composition. While PERMANOVA did not identify significant differences, individual heterogeneity may account for the absence of substantial β-diversity variation, indicating that microbial alterations under study conditions manifest primarily in α-diversity and specific taxon abundances.

(3) Limited Clinical Translation and Causal Inference: Establishment of causality or temporal dynamics is precluded by the cross-sectional design. Models such as LASSO and DLNM demonstrate merely statistical correlations. All associations between weather and microbiota remain correlational. Validation of proposed concepts such as the “weather window” and “therapeutic targets” through large-scale interaction studies, in vivo trials, and longitudinal cohorts remains necessary. These findings cannot yet directly inform clinical treatment decisions.

(4) Absence of Critical Validation Procedures: The seasonal fluctuation patterns and putative biomarker significance of *Escherichia-Shigella* and *Curtobacterium* are based on single-cohort discovery and have not been validated in independent external datasets. The absence of quantitative structural evaluation methods such as histopathology and micro-computed tomography (micro-CT) precludes direct linkage of seasonal microbial variations to specific types of joint structural damage (such as bone density or cartilage integrity). Additional experiments are required to validate underlying biological pathways using metabolomics, germ-free animal models, and host immune biomarkers.

Future research may pursue the following directions:

(1) Integration of Multi-omics and Investigation of Causal Mechanisms: Future investigations should employ longitudinal sample designs encompassing complete seasonal cycles and diverse climatic locations. By integrating metagenomics, metabolomics, and transcriptomics technologies, researchers should quantitatively characterize important metabolites including host immunological biomarkers and short-chain fatty acids. This approach will elucidate causal pathways through which weather influences joint microbiota and functionally validate potential biomarkers. Concurrently, machine learning prediction models should be developed using high-resolution daily real-time meteorological parameters to provide RA patients with precise recommendations for “weather-sensitive” lifestyle interventions.

(2) Germ-free Animal Models and Functional Validation: Strain isolation and culture are necessary to confirm the pathogenic or protective activities of specific seasonally variable microbiota (e.g., *Proteobacteria* enrichment). The effects of specific microbial colonization on joint inflammatory responses and bone loss should be examined in germ-free or antibiotic-treated collagen-induced arthritis (CIA) mouse models. Animal models replicating weather conditions should also be developed. Sequencing technologies should be utilized in conjunction with micro-CT or high-resolution magnetic resonance imaging (MRI) to validate the “weather-microbiome-joint structure” cascade effects. Seasonal variations in osteophyte production, cartilage integrity, and bone density should be monitored simultaneously.

(3) Development of Microbiome-directed Therapeutic Approaches: The interactions between novel nutritional supplements or bioactive peptide treatments and the local joint microbiota should be examined to determine whether these bioactive compounds affect the joint microbiota to produce anti-arthritic benefits. Microbiome-directed adjunctive therapeutic approaches tailored to specific seasonal or climatic characteristics should be thoroughly validated using adjuvant arthritis (AA) models with imaging evaluations and histopathological analysis.

## 4. Materials and Methods

### 4.1. Study Participants

This study enrolled 72 RA patients who attended the outpatient clinic of the Department of Rheumatology and Immunology at the Second Affiliated Hospital of Guizhou University of Chinese Medicine between September 2021 and August 2022. Only patients who were long-term residents of Guiyang City and met the criteria for serious illness were enrolled. All patients satisfied the 2010 European League Against Rheumatism (EULAR) and American College of Rheumatology (ACR) diagnostic criteria for RA [69]. The cohort had a mean age of 45.6 ± 12.0 years, comprising 21 males and 51 females aged 26–64 years. Patients were divided into four groups of 18 patients each: spring (Group A), summer (Group B), autumn (Group C), and winter (Group D). The absence of statistically significant differences (*p* > 0.05) in gender, age, and other characteristics among the 72 patients demonstrated comparability. Refer to the baseline table (Supplementary Materials). Each participant provided written informed consent, and the study was approved by the hospital’s ethics committee. All procedures involving human subjects adhered to the 1964 Declaration of Helsinki and its subsequent amendments.

Exclusion criteria included the following: (1) administration of probiotics or antibiotics within two months of enrollment; (2) local injection of relevant agents into either knee joint within the preceding three months; (3) presence of gastrointestinal symptoms or intestinal infections; (4) concurrent chronic conditions including diabetes mellitus, hypertension, or cardiovascular disease; (5) concurrent autoimmune conditions including OA, SLE, AS or SS; and (6) pediatric patients, pregnant women, lactating women, or individuals with psychiatric disorders.

From 2021 to 2023, data on inpatient admissions were collected from Guiyang First People’s Hospital, the Affiliated Hospital of Guizhou Medical University, and the Second Affiliated Hospital of Guizhou University of Traditional Chinese Medicine, focusing on RA patients who had been long-term residents of Guiyang City. Meteorological data required for calculation of AT, DTR, RH, CC, and MWS were obtained from the National Meteorological Information Center.

### 4.2. Sample Collection

Synovial fluid (minimum 5 mL) was aspirated from the knee joint cavities of study participants under stringent aseptic conditions. Samples were transferred to sterile collection tubes and transported to the laboratory within 30 min. Samples were stored at −80 °C pending analysis.

### 4.3. DNA Extraction and PCR Amplification

Genomic DNA was extracted from synovial fluid using a DNA extraction kit. DNA concentration and purity were assessed using agarose gel electrophoresis and the NanoDrop 2000 spectrophotometer (Thermo Fisher Scientific, Wilmington, DE, USA). Extracted DNA was stored at −20 °C. Bacterial 16S rRNA genes were amplified by PCR using extracted genomic DNA as template, Takara Ex Taq High Fidelity polymerase, and barcoded specific primers. Universal primers 343F (5′-TACGGRAGGCAGCAG-3′) and 798R (5′-AGGGTATCTAATCCT-3′) were employed to amplify the V3–V4 variable region of the 16S rRNA gene for bacterial diversity analysis [70].

### 4.4. Library Preparation and Sequencing

PCR products were identified using agarose gel electrophoresis. Samples were subsequently purified using AMPure XP magnetic beads (Beckman Coulter, Pasadena, CA, USA). Second-round PCR amplification was performed using purified products as templates. Following the second PCR cycle, samples were purified again using magnetic beads. Purified second-round products underwent concentration adjustment and Qubit quantification prior to sequencing. Sequencing was performed on the Illumina NovaSeq 6000 platform (Illumina, San Diego, CA, USA), generating 250 bp paired-end reads. Sequencing services were provided by Shanghai Ouyi Biotechnology Co., Ltd. (Shanghai, China).

### 4.5. Bioinformatics Analysis and Statistical Methods

Library preparation, sequencing, and data analysis were performed by Shanghai Ouyi Biomedical Technology Co., Ltd.; raw data were in FASTQ format. Following data download, primers were initially trimmed from raw data sequences using Cutadapt software (version v4.0). Qualified paired-end raw data were subsequently subjected to quality filtering, denoising, assembly, and de-chimerization using DADA2 [71] and the default parameters of QIIME 2 [72] (version 2020.11), generating representative sequences and ASV abundance tables. Representative sequences for each ASV were selected using the QIIME 2(version 2020.11) software package. Each representative sequence was then annotated by comparison to the Silva database (version 138). Species annotation was performed using the q2-feature-classifier plugin with default parameters.

Differences among the four groups were statistically assessed at the phylum, class, order, family, and genus levels. Both α and β-diversity analyses were performed; α-diversity was measured using the Chao1 index, Observed Species index, Shannon index, and Simpson index. Sample β-diversity was evaluated using Binary Jaccard PCA, and intergroup differences (*p* < 0.05) were identified using PERMANOVA. LEfSe was employed to identify potential biomarker taxonomic units. The Wilcoxon rank-sum test was used to identify differences in abundance characteristics among the four groups at the phylum level. The randomForest package in R was used to generate species importance plots. A DLNM linking RA hospitalization rates to meteorological factors was developed to examine the distinct lagged effects of each meteorological variable on RA patients. Ridge regression and RDA were employed to examine relationships between lagged meteorological data and the α/β-diversity of knee joint effusion microbiota in RA patients.

Statistical analyses of the clinical status of RA patients were conducted using IBM SPSS Statistics for Windows, version 26.0 (IBM Corp., Armonk, NY, USA). The normality of continuous variables was assessed using the Shapiro–Wilk test, and homogeneity of variance was evaluated using Levene’s test. Continuous variables that followed a normal distribution are presented as the mean ± standard deviation (Mean ± SD), and intergroup comparisons were performed using one-way analysis of variance (ANOVA). For continuous variables that did not meet the assumptions of normality, data are expressed as the median with interquartile range [M (P25, P75)], and intergroup comparisons were conducted using the Kruskal–Wallis H rank sum test. Categorical variables are presented as frequencies with percentages [*n* (%)], and intergroup comparisons were performed using the chi-squared (χ^2^) test.

## Figures and Tables

**Table 1:** Common lag window settings for DLNM.

Variable Abbreviation	Select Lag Days	Selection Criteria
AT	Days 0–2	Acute effects [18]
DTR	Days 5	Delayed effects [19]
RH	Days 3–5	Cumulative effects [20]
CC	Days 2–13	Long lag window [19]
MWS	Days 13–14	Long-term transport effects [20]

## Data Availability

The raw data supporting the conclusions of this article will be made available by the authors on request.

## Supplementary Materials

The following supporting information can be downloaded at: https://www.mdpi.com/article/10.3390/ph19030347/s1, Figure S1: (A) ASV classification analysis diagram; (B) Rank-abundance curves; (C) Stacked bar chart of relative abun-dance at the phylum level; Baseline table of clinical information.
